# Aesthetic Alimentation

**Published:** 1909-02

**Authors:** Geo. M. Niles

**Affiliations:** Atlanta, Ga.


### AESTHETIC ALIMENTATION.

BY GEO. M. NILES, M. D., ATLANTA, GA.

"How Near to Good is What is Fair."—*Ben Jonson.*

This title was suggested by a recent editorial in a New York medical journal, arousing the thought that a discussion on the benefits of seeking the beautiful and alluring in eating might be of both interest and profit.

It seems a far cry from John the Baptist, eating locusts and wild honey in the wilderness, to Lucullus, the most princely entertainer of his age; from the rude customs of the Goths in the third century, to the refined doctrines of Omar Khayyam, the material Epicurean of the twelfth century; or from a tramp, with grimy hands, eating a "hand-out" on the back steps, to a Bradley Martin banquet.

These contrasts represent evolution, education and civilization.

But who would gainsay the assertion that John the Baptist would have better enjoyed a tempting meal under a sheltering roof? That the Goths would have been susceptible to some of the amenities of dining? or that the humble tramp would have partaken of his simple repast with greater relish from a clean plate and with clean hands?

Those who eat to live, and those who live to eat are the extremes. They are not representative.

The lunch-counter fiend, who bolts his dinner in five minutes, and the disciple of Horace Fletcher, chewing his food until it is imperceptibly swallowed, are two other extremes. Neither gets the highest pleasure from eating.

It is necessary that we should eat. Fuel must be furnished our bodily furnaces that a proper temperature may be maintained through the vicissitudes of heat and cold. The adult must have enough nourishment to provide energy for the various functions, voluntary and involuntary; while the growing child requires a surplus commensurate with increase in weight.

An active individual cannot care for as much food during an enforced rest, while, in a normal convalescence from wasting disease, the appetite is insistent in its demands.

Now, let us get to the point: As the boy, whistling on his way to school, finds the journey shortened thereby; as the laborer in the field, or the busy housewife in the home finds that singing and cheerfulness lighten the task and speed the hours, so we can apply and elaborate that principle as an aid to that great multitude suffering from capricious appetites and faulty digestion.

The science of the beautiful can deal with actual phenomena, with facts as hard, with rules as fixed and laws as inflexible as do the sciences of biology and physics.

There lies within every intellect, no matter how uncultured, an inherent love for the beautiful. Even the man who can see naught savoring of poetry in "A primrose by a river's brim," has a tuneful chord somewhere within his soul, if it can only be vibrated.

Everybody knows how anger, grief or worry will destroy the desire for food as well as interfere with its digestion.

The writer once saw a cat subjected to an X-ray after being fed. She was quietly stroked until she began to purr, and her stomach could be plainly seen moving in rhythmical waves. In a few moments her tail was pinched, causing a profane outburst of indignation on her part. Immediately all motion of the stomach stopped, nor was it again resumed until she was pacified.

We know how savory odors make the mouth water, and how a tempting array of well-cooked viands suitably garnished pleases the eye; but we do not fully realize how potent the effect on the digestion is the appeal to the aesthetic in our nature.

Under the charming influence of a good meal, tastefully served, where individual fancies are consulted; where there is neither hurry nor tiresome delay; where all the externals that make for beauty are present, and where cheerful conversation rules the board, the digestive organs are at their best. Under these benign influences the vaso-motor nervous system relaxes the arteries furnishing blood to the alimentary tract, a plentiful amount is provided, allowing all the forces that supply our needed energy and heat to perform their functions efficiently

The expensive dinners, embodying culinary symphonies by high-priced chefs, where the aesthetics are represented in every detail, are within the reach of only a few; but to some extent an appeal to the aesthetic in alimentation is within the reach of all.

This fact the writer wishes to stress: that the cultivation of not only the substantial phases of energy and thought, but also the flowers of energy and thought will benefit the mind, the character and also the actual working forces of digestion governed by our sub-consciousness.

A recent writer has said: "There is no tonic so uplifting and renewing as joy, which sets into active exercise every constructive power of the body." So, when suitable food is provided and served, attention to little details which go to make it attractive is time well spent.

Let me insert this simple illustration. Every one will admit that tea served in an egg-shell china cup on a dainty cloth tastes better than if it were in the thick, yellow cup, bearing the scars of rough usage, and poured from a cracked teapot. We may be thirsty and faint, and drink it down from sheer necessity, but something within us revolts, and we do not get the full benefit of it.

The novelists have stirred our emotions with prose; the poets have reached our inner natures with their verses; the painters have uplifted us with their creations of form and color; while the musicians have borne us away on a sea of harmony and melody.

The writer of this article, in an humbler, but fully as important theme, appeals to the senses that lie at the very foundation of our living. He appeals that in furnishing the needed nourishment, it be not shoveled in like coal into a furnace, or poured like corn into a hopper, but that the amenities and aesthetics of civilization be observed. Thus will we not only assist those sub-conscious forces, which work silently and faithfully, to give us their best fruits, a healthy mind in a healthy body; but we will also help onward that evolution of the aesthetic, which is the duty of every enlightened nation.

---

Getting Him Classified.—"What sort of an after-dinner speaker is Bliggins?"

"One of the kind who start in by saying they didn't expect to be called on, and then proceed to demonstrate that they can't be called off."—*Washington Star*.

